# Serum levels of fibroblast growth factor-2 distinguish Takayasu arteritis from giant cell arteritis independent of age at diagnosis

**DOI:** 10.1038/s41598-018-36825-y

**Published:** 2019-01-24

**Authors:** Shoichi Fukui, Ayako Kuwahara-Takaki, Nobuyuki Ono, Shuntaro Sato, Tomohiro Koga, Shin-ya Kawashiri, Nozomi Iwanaga, Naoki Iwamoto, Kunihiro Ichinose, Mami Tamai, Hideki Nakamura, Tomoki Origuchi, Kiyoshi Migita, Yojiro Arinobu, Hiroaki Niiro, Yoshifumi Tada, Koichi Akashi, Takahiro Maeda, Atsushi Kawakami

**Affiliations:** 1 Department of Immunology and Rheumatology, Nagasaki University Graduate School of Biomedical Sciences, Nagasaki, Japan; 2 Department of Community Medicine, Nagasaki University Graduate School of Biomedical Sciences, Nagasaki, Japan; 3 Department of Medicine and Biosystemic Science, Kyushu University Graduate School of Medical Sciences, Fukuoka, Japan; 4 Department of Rheumatology, Saga University, Saga, Japan; 5 Nagasaki University Hospital Clinical Research Center, Nagasaki, Japan; 6 Center for Bioinformatics and Molecular Medicine, Nagasaki University Graduate School of Biomedical Sciences, Nagasaki, Japan; 7 Department of General Internal Medicine and Rheumatology, Clinical Research Center, NHO Nagasaki Medical Center, Omura, Nagasaki, Japan; 8 Department of Rehabilitation Sciences, Nagasaki University Graduate School of Biomedical Sciences, Nagasaki, Japan; 9 Department of Rheumatology, School of Medicine, Fukushima Medical University, Fukushima, Japan; 10 Department of Medical Education, Kyushu University Graduate School of Medical Sciences, Fukuoka, Japan; 11 Department of General Medicine, Nagasaki University Graduate School of Biomedical Sciences, Nagasaki, Japan

## Abstract

Takayasu arteritis (TAK) and giant cell arteritis (GCA) are two major variants of large vessel vasculitis, and age is a major factor in their differential diagnosis. We sought to determine whether the two diseases exist on the same spectrum. We compared the serum levels of multiple cytokines and chemokines in 25 patients with TAK, 20 patients with GCA, and sex- and age-matched healthy donors for either condition (HD-TAK and HD-GCA). To evaluate the effects of age on the levels of cytokines and chemokines, we performed multiple logistic regression analysis using the least absolute shrinkage and selection operator (LASSO) method. The levels of IL-1RA, IL-10, GM-CSF, G-CSF, FGF-2, eotaxin, and IP-10 were significantly different between TAK and GCA, but no differences were found in the levels of IL-6, IL-12(p40), IL-17, IFN-γ, and TNF-α. Significant differences in the levels of IL-1RA, IL-10, GM-CSF, eotaxin, and IP-10 were observed between the HD-TAK and HD-GCA groups. Multiple logistic regression analysis demonstrated that only FGF-2 and IP-10 could significantly distinguish the diseases when added to age. Multiple logistic analysis using factors selected by the LASSO method revealed that FGF-2 was the only significant factor to distinguish the diseases when added to age. Among numerous cytokines and chemokines analyzed, only FGF-2 could be used together with age at diagnosis to differentiate TAK and GCA. Our results suggested the importance of considering the effects of age on serum cytokines.

## Introduction

Large vessel vasculitis (LVV) is characterized as vasculitis that affects the aorta and its major branches. Takayasu arteritis (TAK) and giant cell arteritis (GCA) are characterized as two major variants of LVV by the “2012 Revised International Chapel Hill Consensus Conference Nomenclature of Vasculitides”^1^.

TAK and GCA are considered distinct entities, with the main differences between them being the age at disease onset, clinical features, and distribution of arterial involvement. Namely, TAK typically has a younger age at disease onset, more claudication of extremities, and more aortic involvements compared with those of GCA, which is characterized by older age at disease onset, more headache, and temporal arterial involvements.

However, research evaluating the distribution of aortic involvements based on the findings of magnetic resonance angiography have revealed both strong similarities and subtle differences between TAK and GCA^2^. In addition, some researchers have suggested that TAK and GCA exist on the same spectrum of clinical disease^3,4^. Moreover, it is thought that the histopathologic features of TAK and GCA that demonstrate giant cells^5^ are indistinguishable^1^. As a result, it can be rather hard to draw a fine line between TAK and GCA clinically and histopathologically.

Considering that clinical and histopathological findings may not be fundamentally useful to differentiate the two diseases, age at disease onset has a relatively large role to play in the differential diagnosis. Age at disease onset has traditionally been used to distinguish TAK (=<40 years)^6^ from GCA (>=50 years)^7^. However, age at disease onset also has the potential to engender bias and thereby hamper the diagnosis of TAK or GCA. Furuta *et al*. demonstrated by a latent class analysis using clinical symptoms, laboratory data, and radiological findings that models both with and without age at disease onset were consistent with TAK and GCA^8^. This result suggested that the two diseases were distinctive even when excluding age at disease onset.

In contrast, Maksimowicz-McKinnon *et al*. reported that symptoms, signs, and imaging abnormalities that were characteristic of TAK or GCA were often present in both disorders^3^. Based on this result, they indicated that TAK and GCA might be the same disease, with age-related factors merely influencing the disease expression.

At present, however, there is no definitive answer to the question of whether TAK and GCA have the same pathophysiologic characteristics or exist on the different disease spectrum. One possible method for distinguishing the two diseases is the measurement of multiple cytokines/chemokines. That is, some cytokines have been reported to be associated with TAK^9^ and GCA^10^ based on comparisons to their levels in healthy donors or patients with other diseases.

The objectives of the current study were as follows: (1) to compare cytokines/chemokines levels of TAK with those of GCA at diagnosis before performing any treatments; (2) to evaluate the effect of age on cytokine/chemokine levels; and (3) to reveal disease-specific cytokines/chemokines that are not affected by age in TAK and GCA.

## Methods

### Patients and study criteria

We included patients with TAK and GCA who were registered and followed by Nagasaki University Hospital, Kyushu University Hospital, and Saga University Hospital between March 2003 and July 2017, and who were diagnosed based on the American College of Rheumatology (ACR) 1990 criteria for the classification of Takayasu arteritis^6^ and giant cell arteritis^7^. Of these patients, patients whose sera at the time of diagnosis were available and who had not yet been treated with corticosteroids or other immunosuppressants for TAK and GCA were included in this study. We excluded five patients because they fulfilled both ACR criteria for TAK and GCA concomitantly. Because both ACR criteria do not classify patients with age between older than 40 and younger than 50 as having either TAK or GCA, patients with age between older than 40 and younger than 50 were diagnosed simply based on ACR criteria (each three or more was fulfilled except for age-criterion).

### Healthy donors

We recruited healthy donors (HD) from the staff at Nagasaki University Hospital to compare with TAK patients, and from residents in the town of Saza in Nagasaki prefecture who underwent Specific Health Checkups in 2016 to compare with GCA patients. All HD had no past or present medical histories of inflammatory diseases.

### Ethics approval and consent to participate

This study was performed in accordance with the Declaration of Helsinki and was approved by the Institutional Review Boards of Nagasaki University Hospital (registration number: 15072753), Kyushu University Hospital (registration number: 27-382), and Saga University Hospital (registration number: 2015-08-06). Informed consent for the use of their data was obtained from some of the patients, and an opt-out strategy was chosen for the remainder. The study on Specific Health Checkups residents in the town of Saza was approved by the Ethics Committee for Human Use of Nagasaki University (registration number: 14051404). Written consent forms were obtained from residents in Saza who underwent Specific Health Checkups.

### Clinical, laboratory, and imaging data collection

We accessed the medical records of patients to collect baseline information at diagnosis, including clinical characteristics, laboratory data, data on contrast-enhanced computed tomography and other imaging data. The baseline clinical characteristics at diagnosis included age, sex, symptoms, body mass index (BMI), present smoking status and manifestations at diagnosis. With regard to GCA patients, coexisting polymyalgia rheumatic (PMR) was evaluated based on the 2012 Provisional classification criteria for polymyalgia rheumatic^11^.

The initial laboratory data included C reactive protein (CRP) and the erythrocyte sedimentation rate (ESR). For patients diagnosed with TAK, we included HLA typing. Baseline information at diagnosis was collected within 2 weeks before the start of treatment, except for the HLA typing.

Angiographic classification types for TAK patients^12^ and aortic lesions in GCA patients were evaluated using contrast-enhanced computed tomography (CT). All TAK patients were evaluated by contrast-enhanced CT. Contrast-enhanced CT for GCA patients was also performed when the patient’s attending doctor considered it necessary on the basis of the GCA patient’s clinical characteristics.

### Multiplex cytokine/chemokine bead assays

Multiplex cytokine/chemokine bead assays were performed using diluted serum supernatants and a MILLIPLEX MAP Human Cytokine/Chemokine Magnetic Bead Panel - Premixed 38 Plex (Merck Millipore, Darmstadt, Germany) analyzed with a Bio-Plex® MAGPIX™ Multiplex Reader (Bio-Rad, Hercules, CA) according to the manufacturer’s instructions. The cytokines/chemokines that could be measured by the bead panel included interleukin (IL)-1α, IL-1β, IL-1 receptor antagonist (RA), IL-2, IL-3, IL-4, IL-5, IL-6, IL-7, IL-8, IL-9, IL-10, IL-12 (p40), IL-12 (p70), IL-13, IL-15, IL-17, interferon (IFN)-γ, IFN-α2, growth-related oncogene (GRO), granulocyte-macrophage colony-stimulating factor (GM-CSF), granulocyte colony-stimulating factor (G-CSF), fractalkine, Flt-3 ligand, fibroblast growth factor (FGF)-2, eotaxin, epidermal growth factor (EGF), soluble CD40 ligand (sCD40L), vascular endothelial growth factor (VEGF), tumor necrosis factor (TNF)-β, TNF-α, transforming growth factor (TGF)-α, macrophage inflammatory protein (MIP)-1β, MIP-1α, macrophage-derived chemokine (MDC), monocyte chemotactic protein (MCP)-3, MCP-1, and IFN-γ-inducible protein (IP)-10. The sera of the TAK and GCA patients at diagnosis before any immunosuppressant treatments, including treatments with corticosteroids and HD, were centrifuged within 30 min at 1500 rpm at 4 °C for 5 min, and the liquid phase of the sera was stored at −80 °C until use. Sera from HD were divided into two groups, age- and sex-matched sera from 25 HD for TAK (HD-TAK) and age- and sex-matched sera from 20 HD for GCA (HD-GCA). Sera from HD for TAK were obtained in 2013–2015 and used for Multiplex cytokine/chemokine bead assays in 2015. Sera from HD for GCA were obtained in 2016 and used for Multiplex cytokine/chemokine bead assays in 2017. Some samples of patients experienced maximum one time of the freeze-thawing process and others had no freeze-thawing process before use. Healthy control had no freeze-thawing process before use.

### Statistical analysis

We compared the levels of cytokines/chemokines at diagnosis between TAK and GCA. Categorical variables were described as frequencies and quantitative variables as the median and interquartile range (IQR). The Steel-Dwass test was performed for comparisons of multiplex cytokine/chemokine bead assay results in the four groups, i.e., TAK, GCA, HD-TAK, and HD-GCA. We used two multiple logistic regression model patterns to determine the cytokines/chemokines and age at diagnosis that contribute to the TAK or GCA. In the first pattern, due to the small number of TAK (n = 25) or GCA (n = 20), we used models including age at diagnosis and one variable, respectively. In the second pattern, the multiple model was developed with the use of the least absolute shrinkage and selection operator (LASSO)^13^. A 10-fold cross-validation was used to estimate the penalty parameters in the model. In addition, we used a Firth’s modified logistic regression model to solve a separation problem^14^. A receiver operator characteristic (ROC) curve was described to distinguish TAK from GCA by the levels of FGF-2. All tests were two-sided and a p-value < 0.05 was considered significant. All statistical analyses were performed using JMP Statistical Software, ver. 11 (SAS Institute, Cary, NC), GraphPad Prism ver. 7.0 (GraphPad Software, San Diego, CA), and R version.3.4.3^15^.

## Results

### Patients and clinical characteristics

We excluded 7 patients whose serum samples had been preserved more than 10 years because there was a risk that the cytokines/chemokines in these samples had become unstable and broken down. The final patient groups thus included 25 TAK patients and 20 GCA patients. The demographic, clinical, and laboratory characteristics of patients at diagnosis are shown in Table 1. The median ages at diagnosis were 24 years and 72 years in the TAK and GCA groups, respectively. The median ESR values were 79 mm/h and 72 mm/h in the TAK and GCA groups, respectively. HLA-B52 was found in 63% in TAK patients. Aortic lesions were detected by contrast-enhanced CT in 33% of GCA patients.

**Table 1:** Demographic, clinical and laboratory characteristics of the TAK and GCA patients.

	TAK, n = 25	GCA, n = 20
Age at diagnosis (yrs)	24 (18–38)	72 (67–78)
Females, n (%)	24 (96)	12 (60)
HLA-B52, n (%)	10 (63), n = 16	—
BMI (kg/m2)	18.8 (17.9–22.5)	21.9 (19.2–23.6)
Smoking	5 (20)	4 (20)
Fever	14 (56)	14 (70)
Ophthalmic symptoms	0 (0)	2 (10)
Carotid bruit	16 (64)	—
Discrepancy of BP	16 (64)	—
Pulselessness	6 (24)	—
Cervical pain	7 (28)	—
Chest pain	6 (24)	—
Arthralgia	7 (28)	—
Aortic valve regurgitation	6 (24)	—
New headache	8 (32)	18 (90)
Temporal artery abnormality	^—^	18 (90)
Jaw claudication	^—^	7 (35)
Scalp tenderness	—	8 (40)
Cerebral infarction	—	1 (5)
Coexisting PMR	—	12 (60)
Abnormal artery biopsy	—	3 (50), n = 6
CRP (mg/dL)	5.3 (1.2–11.1)	6.7 (3.6–16.7)
ESR (mm/h)	79 (56–109)	74 (55–93)
Angiographic classification, n (%)	I: 1; IIa: 4; IIb: 7; III: 1; IV: 1; V: 11	—
Aortic lesions in computed tomography	—	5 (33), n = 15

BMI, body mass index; PMR, polymyalgia rheumatica; CRP, C reactive protein; ESR, erythrocyte sedimentation rate.

### Differences in cytokine/chemokine levels between TAK and GCA

**Figure 1:**
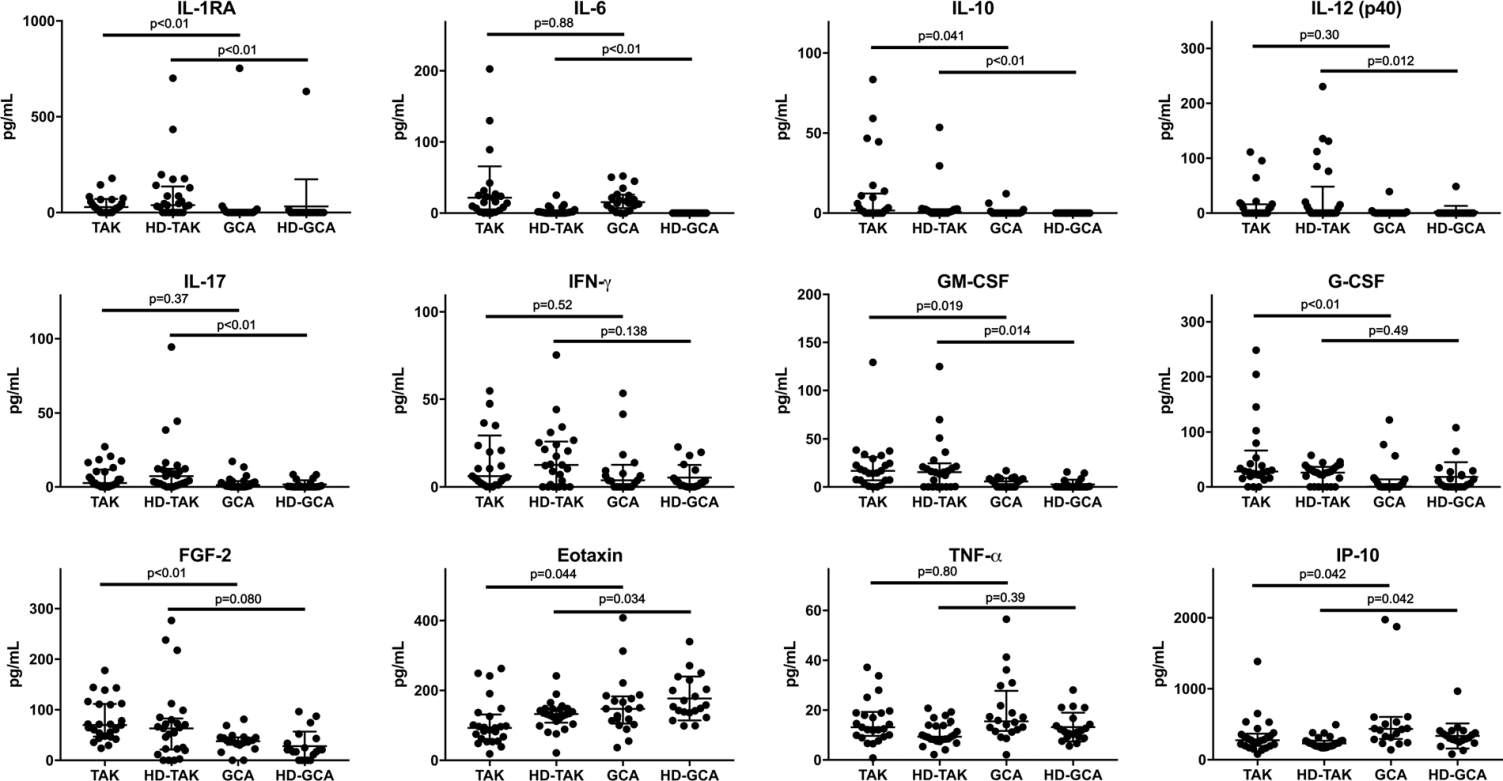
Results of a multiplex cytokines/chemokine bead assay using serum obtained at diagnosis from patients with Takayasu arteritis (TAK) and giant cell arteritis (GCA) and sex- and age-matched healthy donors (HD-TAK and HD-GCA). Symbols represent individual patients or donors. Horizontal lines show the median and interquartile range. The Steel-Dwass test was performed for comparisons of multiplex cytokine/chemokine bead assay results in the four groups, i.e., TAK, GCA, HD-TAK, and HD-GCA.

To determine cytokine/chemokine profiles suitable to distinguish TAK and GCA, we compared the levels of cytokines/chemokines at diagnosis between the two diseases (Fig. 1). Among the 38 cytokines and chemokines tested, the levels of 7 cytokines and chemokines, IL-1RA, IL-10, GM-CSF, G-CSF, FGF-2, eotaxin, and IP-10, were significantly different between TAK and GCA. The levels of IL-1RA, IL-10, GM-CSF, G-CSF, and FGF-2 were higher in TAK than in GCA. The levels of eotaxin and IP-10 were higher in patients with GCA than those with TAK. No differences were found in the levels of IL-6, IL-12(p40), IL-17, IFN-γ, and TNF-α, which have been reported to be associated with TAK^9^ and/or GCA^10^.

### Effects of age on the cytokine/chemokine levels in healthy donors

We allowed up to 9 years in TAK and 12 years in GCA in age-difference in age-matching because TAK and GCA included a 15 and an 86 year-old patient, respectively, and we could not obtain sera from healthy donors whose age completely matched. The median age of 25 HD-TAK and 20 HD-GCA were 29 years old (IQR: 26–31) and 71 years old (IQR: 70–73), and there were no differences between TAK (median: 24; IQR: 18–38) and HD-TAK (p = 0.17) and between GCA (median: 72; IQR: 67–78) and HD-GCA (p = 0.62), respectively. Five of the seven cytokines and chemokines whose levels differed significantly between TAK and GCA, i.e., IL-1RA, IL-10, GM-CSF, eotaxin, and IP-10, also showed significantly different levels between HD-TAK and HD-GCA. These results suggested that age had strong effects on the levels of cytokines and chemokines.

The remaining 2 cytokines/chemokines that were not influenced by age, G-CSF and FGF-2, exhibited significant differences between TAK and GCA without significantly different levels between HD-TAK and HD-GCA [G-CSF: TAK vs. GCA, 28.3 (17.8–66.5) pg/mL vs. 0.3 (0–13.7) pg/mL, p = 0.003, HD-TAK vs. HD-GCA, 26.2 (0–36.5) pg/mL vs. 8.7 (0–26.3) pg/mL, p = 0.49; FGF-2: TAK vs. GCA, 70.0 (47.1–111.6) pg/mL vs. 38.0 (30.8–45.4) pg/mL, p < 0.001, HD-TAK vs. HD-GCA, 63.2 (21.3–82.7) pg/mL vs. 21.2 (2.8–40.8) pg/mL, p = 0.080].

### Multiple logistic regression analysis of the age at diagnosis and each cytokine/chemokine

We performed multiple logistic regression analysis of age at diagnosis and each cytokine/chemokine whose level was significantly different between patients with TAK and GCA to determine factors contributing to the diagnosis of TAK or GCA. FGF-2 and IP-10 were factors that significantly contributed to whether the diagnosis was TAK or GCA when added to age at diagnosis (FGF-2: p = 0.0020; IP-10: p = 0.0317; Table 2).

**Table 2:** Logistic regression analysis of GCA to TAK.

Variable	Odds ratio (95%CI)	p-value
Age at diagnosis	1.213263 (1.098749–1.505406)	<0.0001
IL-1RA	1.004395 (0.998304–1.013413)	0.1568
Age at diagnosis	1.203089 (1.093787–1.444249)	<0.0001
IL-10	0.719267 (0.478324–1.022482)	0.0682
Age at diagnosis	1.207694 (1.095947–1.51713)	<0.0001
GM-CSF	1.003723 (0.999669–1.033019)	0.0842
Age at diagnosis	1.173548 (1.083717–1.372381)	<0.0001
G-CSF	1.00381 (0.974873–1.015528)	0.6795
Age at diagnosis	1.231522 (1.100563–1.570795)	<0.0001
FGF-2	0.914779 (0.771112–0.978033)	0.0020
Age at diagnosis	1.180993 (1.086083–1.41796)	<0.0001
Eotaxin	1.01042 (0.997674–1.031118)	0.1132
Age at diagnosis	1.230264 (1.096659–1.638509)	<0.0001
IP-10	1.003884 (1.000169–1.014025)	0.0317

IL: interleukin; IL-1RA: IL-1 receptor antagonist; GM-CSF: granulocyte macrophage colony-stimulating factor; G-CSF: granulocyte colony-stimulating factor; FGF-2: fibroblast growth factor-2; IP-10: IFN-γ-inducible protein-10.

### The multiple model using the LASSO method

We used a multiple model with the LASSO method to determine factors contributing to a diagnosis of TAK or GCA, because we had too few patients to perform a multiple logistic regression analysis using all 38 cytokines/chemokines. Eotaxin, FGF-2, fractalkine, G-CSF, IL-2, IL-9, TNF-α, VEGF, and sCD40L were extracted as contributing factors. Because the conventional multiple logistic model could not obtain the convergence—i.e., there was a separation problem—we used Firth’s logistic regression model. As a result, age at diagnosis and FGF-2 were significant (Table 3).

**Table 3:** Logistic regression analysis using LASSO of GCA to TAK.

Variable	Odds ratio (95% confidence interval)	P value
Age at diagnosis	3.1818 (1.3187–27.0747)	0.0074
Eotaxin	1.2516 (0.4170–4.8811)	0.6264
FGF-2	0.3159 (0.0529–0.8476)	0.0205
Fractalkine	1.5041 (0.5807–5.3921)	0.3260
G-CSF	0.9044 (0.1257–2.7458)	0.8418
IL-2	0.7740 (0.1127–5.6015)	0.6858
IL-9	1.7561 (0.7386–10.6566)	0.2171
TNF-α	1.0077 (0.2819–3.7319)	0.9874
VEGF	1.7885 (0.7027–12.2498)	0.2049
sCD40L	0.7150 (0.1108–2.2897)	0.5488

FGF-2: fibroblast growth factor-2; G-CSF: granulocyte colony-stimulating factor; IL: interleukin; TNF-α: tumor necrosis factor-α; VEGF: vascular endothelial growth factor; sCD40L: soluble CD40 ligand.

### The cutoff level of FGF-2 to distinguish TAK from GCA

**Figure 2:**
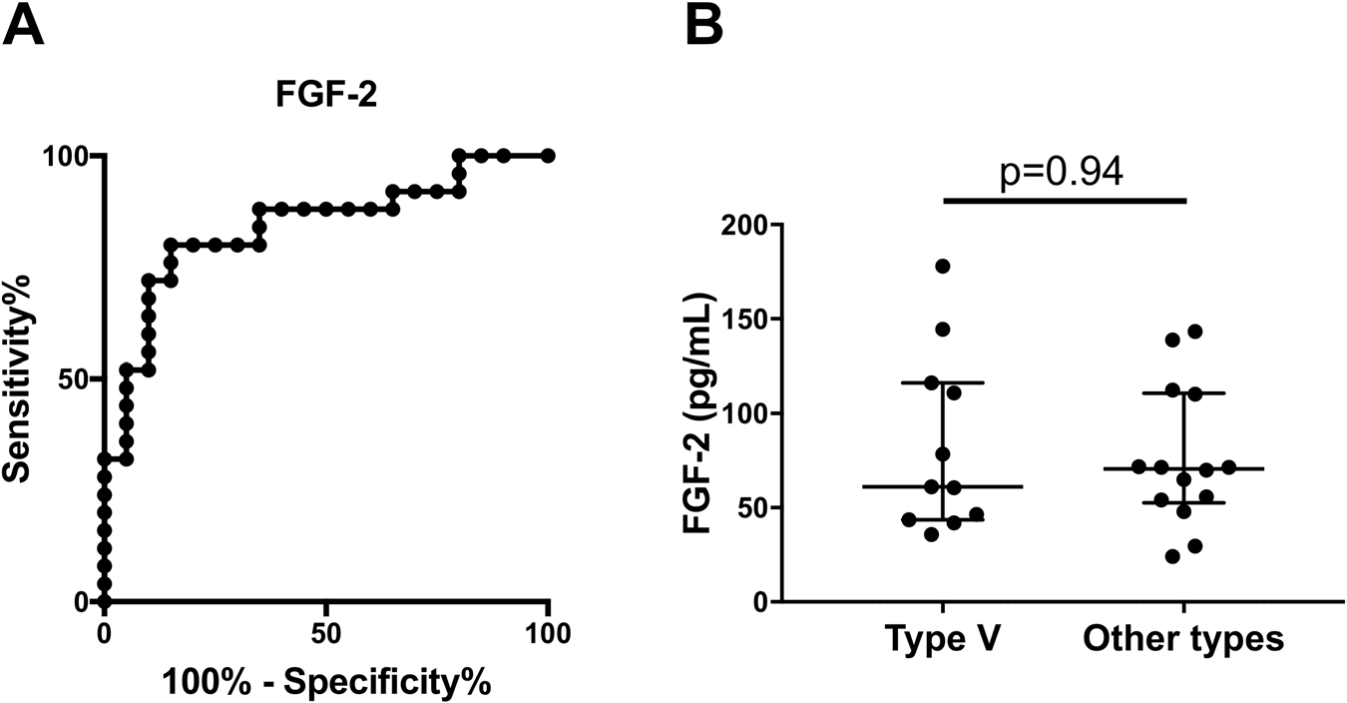
(**A**) An ROC curve for fibroblast growth factor-2 (FGF-2) to distinguish TAK from GCA (cutoff level: 46.4 pg/mL, Sensitivity: 80%, specificity: 85%, area under the curve: 0.84). (**B**) Comparison of levels of FGF-2 between type V of TAK and other types of TAK (Type V: 61.1 (43.7–116.2) pg/mL vs. Other types: 70.7 (52.5–110.7) pg/mL, p = 0.94).

We determined the sensitivity and specificity of FGF-2 to distinguish TAK from GCA by describing an ROC curve (Fig. 2A). The cutoff value of FGF-2 was 46.4 pg/mL, yielding sensitivity and specificity values of 80% and 85%, respectively. The area under the ROC curve (AUC) for the FGF-2 levels was 0.84.

### The effects of angiographic classification types for TAK patients to levels of FGF-2

We evaluated the effects of the extent of the disease defined by angiographic classification to levels of FGF-2. We divided patients with TAK into two groups, the group of Type V and the group of other types excluding Type V. We detected no differences between the two groups (p = 0.94, Fig. 2B).

## Discussion

Our results showed the following in regard to cytokine/chemokine levels in patients with TAK and GCA and their relation to age. (1) There were significant differences in the levels of IL-1RA, IL-10, GM-CSF, G-CSF, FGF-2, eotaxin, and IP-10 between the TAK and GCA groups. Considering differences between HD-TAK and HD-GCA, however, only G-CSF and FGF-2 appeared to have meaning. (2) FGF-2 and IP-10 were the only factors to significantly distinguish the two diseases when added to the age at diagnosis by multiple logistic regression analysis of the age at diagnosis and each cytokines/chemokines. (3) The multiple model using the LASSO method revealed that FGF-2 was the only factor to significantly distinguish the two diseases. Based on all of these results, we consider that FGF-2 is associated with the pathophysiologic differences between the two diseases when considering the effects of age to the levels of cytokines/chemokines.

Different methods have been used to investigate the role of cytokines/chemokines in TAK and GCA^9^, including detection of the serum and plasma levels of these markers, and quantification of the protein expression in or from producing cells either in circulating/cultured cells or arterial specimens and messenger RNA. Based on their analysis using the culture supernatants of stimulated peripheral blood mononuclear cells and aortic inflammatory infiltrates, Saadoun and Garrido *et al*. suggested that the Th1 and Th17 pathways, such as overproduction of IL-2, IFN-γ, TNF-α, IL-17A, contribute to the systemic and vascular manifestations of TAK compared with GCA^16^. Higher levels of cytokines involved in Th17 response is also associated with extensive arterial involvements of TAK even in remission^17^. In addition, serum IL-6 has been reported to be a potential biomarker of TAK^9^. In GCA patients, however, Th17 dominance in the early phase, Th1 persistence in the chronic phase^18^ and elevated serum IL-6 levels have also been demonstrated^10^. However, the limited data from serum biomarkers by multiplex cytokine/chemokine bead assays in the present study did not reveal a clear difference among Th1-related cytokines, Th17-related cytokines and IL-6 between TAK and GCA. Since we examined neither the flow cytometric results of circulating/cultured cells nor pathological findings of arterial specimens, further precise investigations will be needed before drawing any definitive conclusions in regard to the T helper cell response and its role in patients with TAK and GCA.

It is also important to consider age when comparing these two diseases, because there is a problem regarding immunosenescence. It has been reported that aging was associated with an increase in cellular Th2 bias and a decline in total numbers of T cells in healthy blood donors^19^. Alvarez-Rodriguez *et al*. reported that aging had a negative impact on the circulating levels of IL-17^20^. In consideration of these reports, we added HD-TAK and HD-GCA groups to the present analysis of differences between TAK and GCA, and our results suggested an unexpected phenomenon in regard to Th1 and Th17.

FGF-2 is a heparin-binding growth factor which has pleiotropic roles as a mitogenic, angiogenic, and survival factor involved in cell migration, cell differentiation, and a variety of developmental processes^21^. Along with the vascular endothelial growth factors, FGF-2 is one of the most important factors in angiogenesis^22^. It was reported that age and sex had no effect on the serum level of FGF-2^23^. Kaiser *et al*. reported that the expression of FGF-2 in biopsy specimens of the temporal arteries of GCA patients had no correlation with the neovascularization in temporal arteries^24^. To the best of our knowledge, the present study was the first to report a higher level of FGF-2 in patients with TAK compared with patients with GCA. The higher level of FGF-2 might be the result of the longer and wider aortic and arterial lesions in TAK compared to GCA.

We also found that the levels of two factors differed between TAK and GCA—namely, G-CSF was higher in patients with TAK and IP-10 was higher in those with GCA. It has been reported that G-CSF induced endothelial cells to migrate and proliferate^25^ and stimulate angiogenesis^26^. IP-10 has been reported to inhibit fibroblast growth factor-induced neovascularization^27^. Based on these reports, TAK might have higher angiogenic activity than GCA.

In addition to the difference of methodology compared with previous studies, our study had another important limitation. That is, we did not account for the concept of cranial GCA (C-GCA) and large vessel GCA (LV-GCA). LV-GCA has been reported to have a lower rate of vision loss, higher relapse rate, and greater corticosteroids requirements^28^. However, although they might be distinct entities of GCA, we had no data of aortic lesions in computed tomography of five patients with GCA and we had too few patients to assess the effects of C-GCA and LV-GCA.

As another limitation, we may underestimate the discriminating values of IL-1RA, IL-10, GM-CSF, G-CSF, eotaxin, and IP-10 except for FGF-2 because of the strong effect of age at diagnosis to the differentiation of TAK and GCA. Simply using these cytokines/chemokines to differentiate TAK from GCA may be possible if paying attention to patients with atypical age at diagnosis, especially elderly-onset TAK.

Given these limitations, we need to pay attention to the clinical utility of serum FGF-2 for TAK and GCA. Because HD-TAK have the same levels of FGF-2 as those of TAK patients shown in Fig. 1, FGF-2 cannot be used to distinguish TAK from healthy persons. In addition, because we used ACR criteria to diagnose TAK and GCA, age had a huge impact to distinguish them. Therefore, the diagnostic clinical utility of serum FGF-2 needs to be confirmed in a cohort of large vessel vasculitis including LV-GCA and elderly-onset TAK.

In conclusion, our analysis of the serum levels of multiple cytokines/chemokines demonstrated that the levels of FGF-2 were sufficiently different to distinguish between TAK and GCA when considered together with age at diagnosis. Based on our results, it is difficult to differentiate these diseases based on the serum levels of cytokines related to Th1 and Th17 cells. Evaluation of age is thus vital to assess cytokines and chemokines correctly.
